# Modeling of Chromium, Copper, Zinc, Arsenic and Lead Using Portable X-ray Fluorescence Spectrometer Based on Discrete Wavelet Transform

**DOI:** 10.3390/ijerph14101163

**Published:** 2017-09-30

**Authors:** Fang Li, Anxiang Lu, Jihua Wang

**Affiliations:** 1 Beijing Research Center for Agricultural Standards and Testing, Beijing Academy of Agriculture and Forestry Sciences, Beijing 100097, China; viki2069@126.com (F.L.); jhwangatfm@163.com (J.W.); 2 Beijing Municipal Key Laboratory of Agriculture Environment Monitoring, Beijing 100097, China; 3 Risk Assessment Lab for Agro-products (Beijing), Ministry of Agriculture, Beijing 100097, China

**Keywords:** X-ray fluorescence, heavy metal, soil, wavelet transform

## Abstract

A modeling method based on discrete wavelet transform (DWT) was introduced to analyze the concentration of chromium, copper, zinc, arsenic and lead in soil with a portable X-ray fluorescence (XRF) spectrometer. A total of 111 soil samples were collected and observed. Denoising and baseline correction were performed on each spectrum before modeling. The optimum conditions for pre-processing were denoising with Coiflet 3 on the 3rd level and baseline correction with Coiflet 3 on the 9th level. Calibration curves were established for the five heavy metals (HMs). The detection limits were compared before and after the application of DWT, the qualitative detection limits and the quantitative detection limits were calculated to be three and ten times as high as the standard deviation with silicon dioxide (blank), respectively. The results showed that the detection limits of the instrument using DWT were lower, and that they were below national soil standards; the determination coefficients (*R^2^*) based on DWT-processed spectra were higher, and ranged from 0.990 to 0.996, indicating a high degree of linearity between the contents of the HMs in soil and the XRF spectral characteristic peak intensity with the instrument measurement.

## 1. Introduction

Rapid population growth and urbanization, together with expansion of industrial production, have resulted in serious soil contamination issues globally. Heavy metal (HM) pollution is a major type of soil pollution. HMs in soil mainly derive from atmospheric dust, sewage irrigation, mining and smelting, and the application of pesticides and fertilizers [1]. Heavy metal pollution in soil deteriorates air and water quality, causes a decline in the yield and quality of crops, and threatens human health through the food chain [2]. HM pollution is difficult to identify because of the range of different contaminants and attempts to conceal pollution events, and difficult to remediate owing to the complex chemical behavior of HMs and their ecological effects [3]. The main detection methods of HMs in soil involve the use of strong acid to digest soil samples, which are then tested by methods such as atomic absorption spectroscopy (AAS) [4], inductively coupled plasma atomic emission spectrometry (ICP-AES) [5], and inductively coupled plasma mass spectrometry (ICP-MS) (ICP-MS) [6]. These methods are highly accurate and have good precision; however, the pre-processing steps are tedious, time-consuming, expensive, and the strong acid used in the experiments may also contribute to contamination. A rapid detection method with simple pre-processing and accurate detection results is needed. X-ray fluorescence (XRF) spectroscopy has the potential to meet these requirements, owing to its ability to rapidly analyze samples, the simplicity of its sample pre-treatment and operation, its high sensitivity, the wide range of elements analyzed, its low cost, and its ability to perform in-situ testing [7,8,9]. Furthermore, chemical methods have much lower detection limits, but also require that the digestion is complete and that there are no errors in the dilutions, which leads them to sometimes become more imprecise and inaccurate than the spectrometry of XRF, which requires virtually no pretreatment of the sample.

Some researchers have studied the factors influencing modeling results with portable XRF spectrometers [10,11,12,13]. XRF has been applied to the detection of HM concentration in many studies [14,15,16,17,18]; however, little attention has been paid to modeling and pre-processing of the spectral data, which is required in order to improve the accuracy of XRF test results. The composition of soil is complex, resulting in matrix effects when using XRF for analysis. Variations of the sample composition and physical/chemical properties will affect the fluorescence enhancement effect, the intensity of XRF, the limits of detection and the absorption of primary X-rays and XRF [19,20]. Thus, it is necessary for the instrument developers to remove and optimize the matrix effects, and improve the accuracy and precision of instruments. For these purposes, it is essential to establish accurate models for calibration curves. This paper concerns the testing and processing of large numbers of soil samples to obtain a regression model for HMs. Eight kinds of toxic HMs in soil—cadmium, chromium, copper, zinc, lead, arsenic, nickel and mercury—have had their limited concentration specified by the Environmental Quality Standard for Soils, GB15618-1995. It’s difficult to quantify the concentration of cadmium, nickel and mercury with XRF due to their unique characteristics; the modeling is not satisfactory. So the other five kinds of HMs were chosen to be analyzed in this paper.

Measured spectra are often accompanied by high-frequency noise and low-frequency baseline interference, which are the main factors affecting spectral identification. Denoising and baseline removal directly affect the quality of spectral analysis. The wavelet transform (WT) [21] developed in recent years is a signal analysis tool based on the time-frequency domain that has advantages including good time-frequency localization, flexibility of base choice, and decorrelation, making it a superior method for filtering noise and removing the baseline of spectral data.

In this paper, a denoising and baseline correction method using discrete wavelet transform (DWT) is proposed to achieve more accurate detection results before data analysis. This is the first time that DWT has been used as a pre-processing algorithm in XRF spectroscopy for soil detection. Both the raw spectral data and processed data under DWT were used in modeling the HMs. Our results show that the determination coefficients (*R^2^*) were improved, and the detection limits were reduced, meaning that the accuracy of the modeling was successfully improved. Thus, we show that DWT is an efficient method for processing XRF spectral data before modeling.

## 2. Materials and Methods

### 2.1. Collection of Soil Samples, Equipment and Measurement Conditions

**Figure 1:**
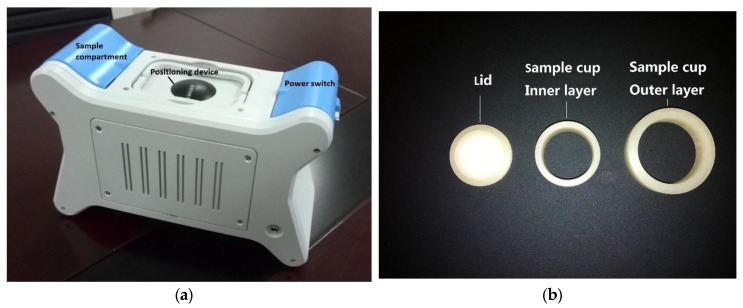
XRF spectrometer and sample cup used for detection in this study: (**a**) XRF spectrometer; (**b**) sample cup. The spectrometer can be used both in laboratory and in the field, linked with a laptop.

All measurements were made with a portable XRF spectrometer (SX-100S, Beijing Research Center for Agricultural Standards and Testing, Beijing, China) fitted with an Ag anode X-ray tube, Al + Mo filter, and silicon drift detectors (Figure 1). The instrument was operated at a voltage of 30 kV, current of 30 μA, and detection time of 100 s. Soil samples were placed into an ethylene sample cup (D × H: 30 mm × 10 mm, NCS Testing Technology Co., Beijing, China) with a fixed Mylar film (Premier Lab Supply Co., Woburn, MA, USA; special film for X-ray analysis, thickness: 6 μm) collar.

A total of 111 samples were collected, including 46 national standard soil samples (National Standard Material Research Center, Beijing, China), 30 standard addition soil samples (collected from Heilongjiang, Yunnan, Jiangsu and Xinjiang provinces from typical soil, namely, black soil, paddy soil, red soil, and brown soil), and 35 natural soil samples (collected from around Beijing) from the soil surface of farmland at a depth of 0–20 cm. The samples were preserved after air-drying, ground, and sieved with a nylon mesh indoors. The standard additional soil samples were mixed with the appropriate metal salts over a range of concentrations by serial dilution. All the tools used during the processing were made of ceramic or agate to ensure no interfering XRF signals at the tested heavy metal peak energies. Analysis of the HMs in the soil samples was performed in accordance with national standards, including the national standard soil samples (GSS-4) for quality control; three spectra were collected for each sample. The content of chromium (Cr), copper (Cu), zinc (Zn), lead (Pb) were determined with a Solaar-M (Thermo Fisher Scientific Inc., Waltham, MA, USA) atomic absorption graphite furnace. Arsenic (As) content was tested with a AFS-830 (Jitian Instrument Inc., Beijing, China) atomic fluorescence analyzer.

### 2.2. Spectra Processing

#### 2.2.1. Wavelet Transform

Wavelet transform [22] is a localized analysis of time (space) frequency by stretching shift operation on the signal (function) multiscale refined gradually, and ultimately achieves time segmentation at high frequencies and frequency segmentation at low frequencies [23,24]. Since most signals in nature are non-stationary, the characteristics of WT make it superior for signal analysis compared to Fourier transform and short-time Fourier transform. Fourier transform can only capture the frequency components contained in a signal, but cannot calculate when the components appear, so it is inherently defective for dealing with non-stationary signals. Short-time Fourier transform (STFT) adds windows during the decomposition process to obtain time components, but it is hard to find the appropriate width of the window when dealing with non-stationary signals. The narrow window has a high time resolution and a low frequency resolution, the wide window has a low time resolution and a high frequency resolution. For time-varying non-steady signals, high frequencies are suitable for small windows, low frequencies are suitable for large windows. However, the STFT window is fixed and the width does not change during one STFT calculation. So STFT is still unable to meet the need to analyze the frequency change of an unsteady signal. The WT can automatically adapt to the requirements of time-frequency signal analysis, and thus to focus on any details of a signal. The wavelet is a function family produced by panning or stretching a form function ψ(t) that satisfy certain conditions [25]:

(1) $$\psi_{a,b}\left(t\right)=\frac{1}{\sqrt{a}}\psi \left(\frac{t-b}{a}\right)$$

where ψ(t) is the wavelet basis (WB), and a and b are the scale and translation parameters, respectively. Many wavelets have been developed, including Haar, Daubechies, Coiflet, Symlets, Mexican Hat. The DWT is often used instead of WT in computation by Equation (2) [26].

(2) $$\psi_{j,k}\left(t\right)=2^{-\frac{j}{2}}\psi \left(2^{-j}t-k\right)$$

where  j,k∈Z. The discrete wavelet coefficients of a function f(t) or a signal are computed according to Equation (3) [27].

(3) $$C_{j,k}=<f,\psi_{j,k}>\;=\int_{-\infty }^{\infty }f\left(t\right)\psi_{j,k}^{*}\left(t\right)dt$$

#### 2.2.2. Mallat Algorithm

**Figure 2:**
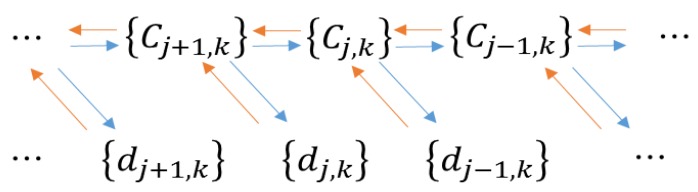
Mallat decomposition and reconstruction algorithm. Directions of blue and red arrows indicate the decomposition and reconstruction algorithms, respectively.

The Mallat [28] algorithm is often performed for DWT. Combined with multi-resolution analysis, Mallat proposes a method with a sub-band structure to achieve a DWT algorithm, unified computing sub-band filter and WT. The *L*^2^(*R*) space produces two subspaces with multi-resolution analysis (MRA)—scale space {*V_j_*}*_j_*_∈Z_ and wavelet space {*W_j_*}*_j_*_∈*Z*_—and {*ϕ_j,k_*}*_j,k_*_∈*Z*_ and {ψ*_j,k_*}*_j,k_*_∈*Z*_ are the orthonormal bases of the two spaces, respectively. The process of the Mallat decomposition and reconstruction algorithm is shown in Figure 2. It is assumed that a conjugate mirror filter is produced by the orthogonal scaling function and wavelet function [29,30]. The scaling coefficients and wavelet coefficients of the WT as *c_j,k_* and *d_j,k_*, and the recursive formulas are shown in Equations (4) and (5), which enable the calculation {*c_j,k_*, *d_j,k_*} [31]. Thus, with the initial sequence {*c_j,k_*}*_k_*_∈Z_ in space *V_j_*, we can calculate all the scale coefficients and wavelet coefficients of any spaces *V_j_* (j < J). Equations (4) and (5) are known as the DWT decomposition formula. The Mallat reconstruction formula is given in Equation (6):

(4) $$c_{j,k}=<f\left(t\right),\varphi_{j,k}>=\frac{1}{\sqrt{2}}\underset{n\in Z}{\sum }h_{n}c_{j+1,n+2k}$$

(5) $$d_{j,k}=<f\left(t\right),\psi_{j,k}>=\;\frac{1}{\sqrt{2}}\underset{n\in Z}{\sum }g_{n}c_{j+1,n+2k}$$

(6) $$c_{j+1,k}=<f\left(t\right),\varphi_{j+1,k}\geq =\frac{1}{\sqrt{2}}\underset{n\in Z}{\sum }\left(h_{n-2k}c_{j,k}+g_{n-2k}d_{j,k}\right)$$

#### 2.2.3. Wavelet Transform Processing

Wavelet denoising mainly includes three steps: Signal decomposition, high-frequency coefficient threshold quantization and signal reconstruction. The most critical step in the process is how to select and quantify the threshold, which directly relates to the quality of denoising.

### 2.3. Determination and Validation of Calibration Curves

The characteristic Kα X-ray line, peak position and absorption band of five HMs are listed in Table 1. After processed by DWT, calibration curves were constructed with mean values of counts and standard values. The measured spectra were re-transferred back to the software to obtain detection values calculated from the calibration curves. Detection values and standard values were contrasted to verify the accuracy of the instrument. To determine the detection limits of the instrument and investigate the reproducibility and accuracy further, soil samples with different compositions together with silicon dioxide (blank, Aladdin industrial Co., Shanghai, China) were chosen for retesting. Each sample was tested 11 times.

## 3. Results and Discussion

### 3.1. WT Processing Results

#### 3.1.1. Evaluation Criteria of Denoising Results

To ensure the consistency between the denoising and original spectrum, we examined the signal to noise ratio (SNR, Equation (7)), mean square error (MSE, Equation (8)) and information entropy (H, Equation (9)) [32,33]. For SNR and H, a larger value is better, for MSE a lower value is better.

(7) $$\mathrm{SNR}=10\times \log\overset{N}{\underset{1}{\sum }}\frac{y_{i}^{2}}{{\left(x_{i}-y_{i}\right)}^{2}}$$

(8) $$\mathrm{MSE}=\frac{1}{N}\overset{N}{\underset{i=1}{\sum }}{\left(y_{i}-x_{i}\right)}^{2}$$

(9) $$H=-\overset{N}{\underset{1}{\sum }}p\left(x_{i}\right)\times \log\left(2,p\left(x_{i}\right)\right)$$

where *N* is the channel number, *y_i_* is the original value, *x_i_* is the noise-free value after processing, *p(x_i_)* is the probability of information appears at a certain point. To comprehensively evaluate the effect of denoising, the coefficient α was created, which is proportional to the denoising effect:

(10) $$\alpha =\frac{SNR\times H}{MSE}$$

#### 3.1.2. Selection of WB

All the signal processes were performed on a desktop (Hewlett-Packard, P6-1499CN, Palo Alto, CA, USA) using MATLAB version 2014a software (MathWorks Inc., Natick, MA, USA). All programs for noise reduction and baseline correction were written locally in the lab. Some commonly used wavelets were chosen to denoise the XRF signal at a decomposition level of 4 with soft thresholding mode (Table 2, Coiflet (coif) 2–5, Daubechies (db) 5–10, Symmlet (sym) 5–8). Coif 3 was chosen as the optimized wavelet and given the lowest value of *α*. The optimal decomposition level was determined by coif 3 from level 3 to 10 (Table 3). Level 3 proved to be the optimum.

**Figure 3:**
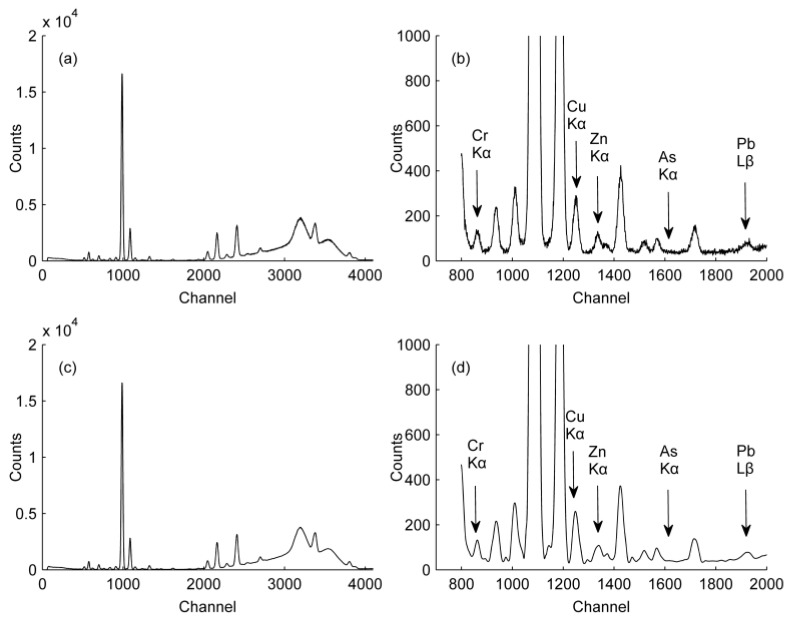
Denoising results with coif 3 at level 3: (**a**) Original signal; (**b**) Enlarged view of (**a**); (**c**) Denoised signal; (**d**) Enlarged view of (**c**).

The detected spectra of 111 samples were processed by DWT. To show the results clearly, a representative spectrum is shown in Figure 3. The denoising results in the figure in the channel range 700–2000 are expanded to enable observation of signal processing effects on the HMs peaks. The peaks of Cr, Cu, Zn, As, and Pb appear around the channels 816–856, 1224–1264, 1315–1355, 1609–1649, and 1926–1969, respectively. Figure 3c,d show that with coif 3 at level 3, the noise level was successfully reduced without affecting the peak shape.

To effectively resolve the spectrum and improve the operation speed of the algorithm, a “speed-up” method of peeling peaks was used. The wavelet decomposition was used for the XRF spectrum *f*^0^(n) to extract the approximate part *f^j^*(*n*) from a higher scale; if *f*^0^(*n*) > *f^j^*(*n*), then *f*^0^(*n*) = *f^j^*(*n*). In this way, peak pealing was accelerated; the same process was then applied to the new *f*^0^(*n*), and the cycle was repeated several times, in order to obtain a relatively gentle curve. Wavelet decomposition was used on the relatively flat curve, and extracted an appropriate level as an approximation of the XRF spectra baseline to achieve better results.

**Figure 4:**
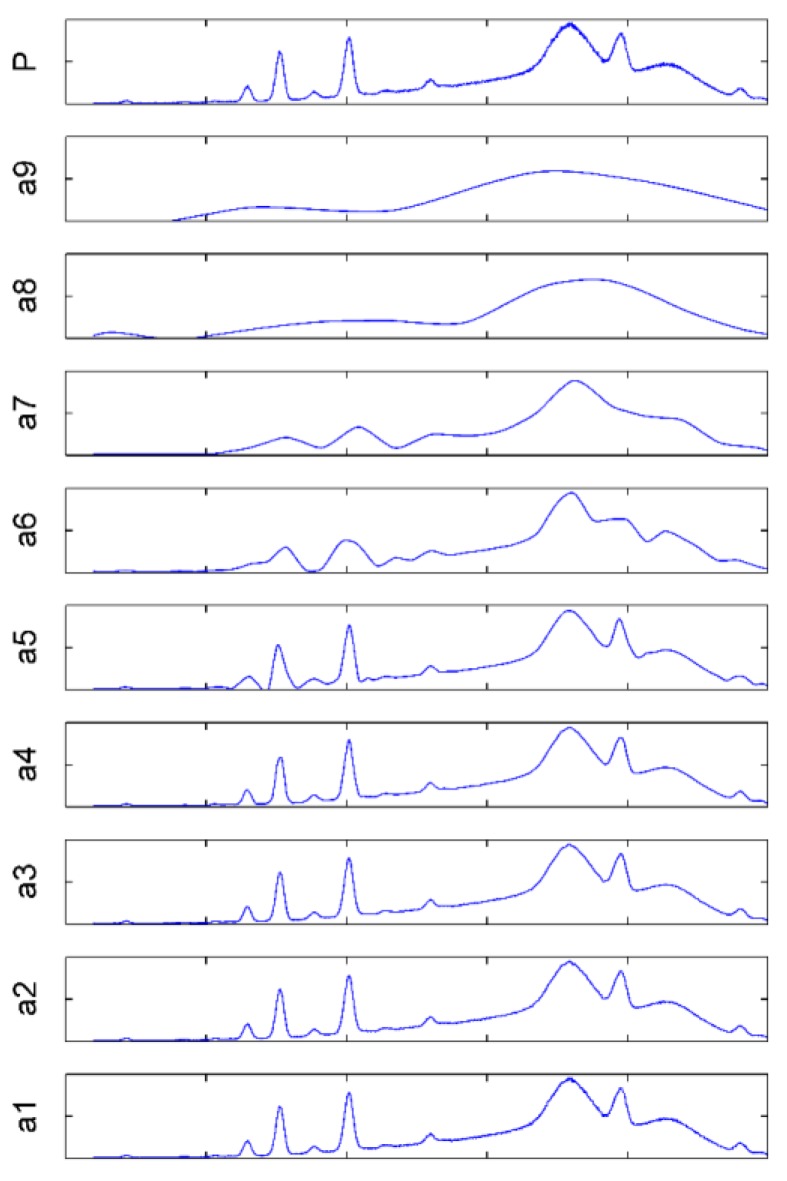
Approximate background of XRF obtained by DWT with the coif 3 at level 9. P indicates the raw spectrum, a1–a9 are results on the decomposition levels 1–9.

**Figure 5:**
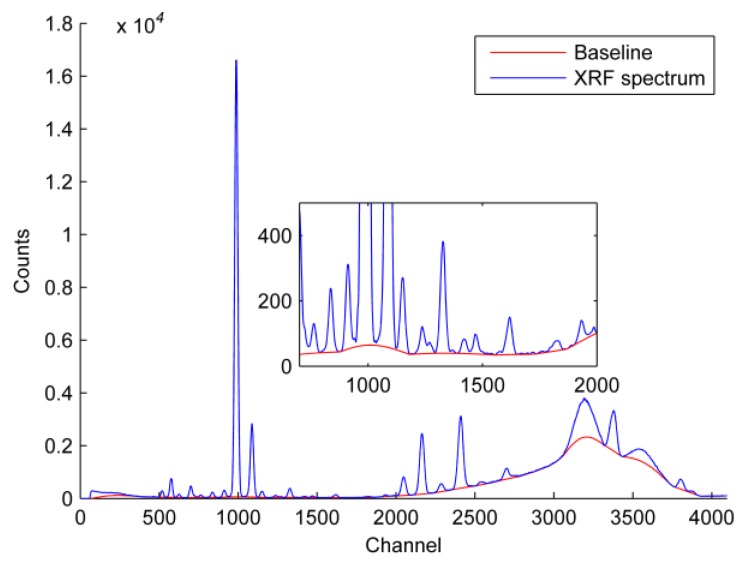
Baseline correction after denoising with DWT. Inset shows a spectrum enlarged for 800–2000 channels.

Through many experiments, the optimized decomposition level was 9, which achieved a good approximation of the baseline (Figure 4), and the decomposition result was shown in a9. The spectrum between 1500–4000 channels was intercepted to show the result of calculation by DWT clearly. The signal at a9 was regarded to be the baseline, and was used for background deduction. Baseline corrections (coif 3 at level 9) were performed based on denoising (coif 3 at level 3). The baseline was successfully removed without losing peak information (Figure 5). The spectrum between 800–2000 channels was magnified to show the calculation’s effects on the HM peaks more clearly; the baseline and peak positions were unaffected by the processing.

### 3.2. Instrument Calibration Curves

**Figure 6:**
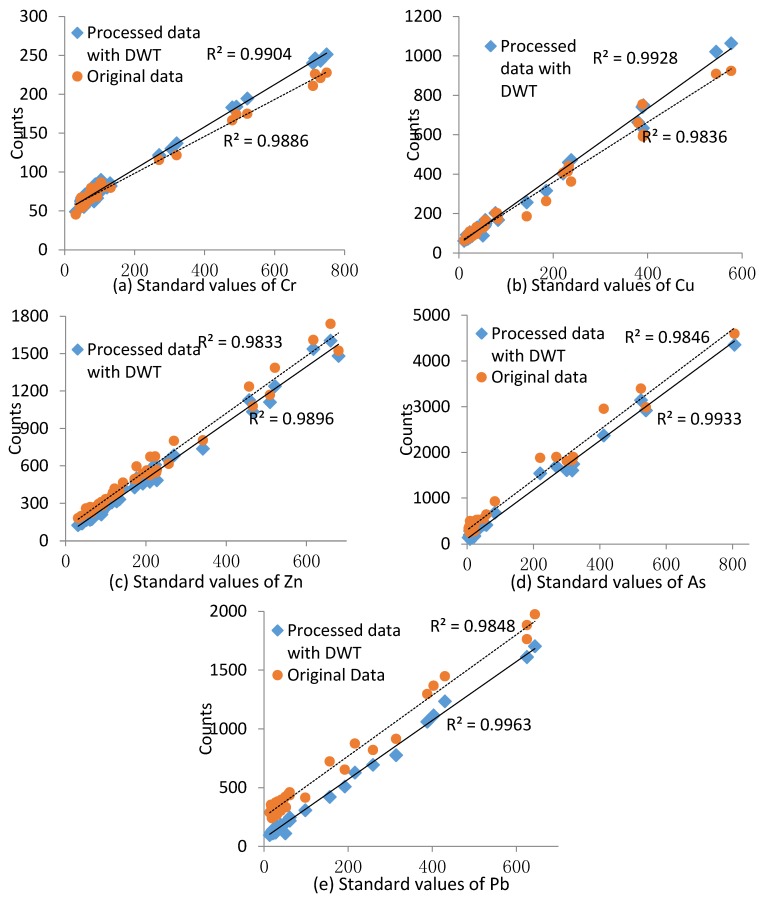
Calibration curves for Cr (**a**), Cu (**b**), Zn (**c**), As (**d**) and Pb (**e**). Spectral data were pre-processed with DWT.

The concentration ranges of Cr, Cu, Zn, As, and Pb were 32–749, 11.4–577, 31–680, 4.4–806, and 13.4–644 mg/Kg. The calibration curves were drawn with the raw spectral data and processed spectral data, which were obtained from each of the processed lines by the wavelet analysis using the method described in 2.3. Comparisons were made between the modeling results with the data before and after processing. The values of *R^2^* for Cr, Cu, Zn, As, and Pb based on the raw spectral data were 0.9886, 0.9836, 0.9833, 0.9846 and 0.9846, respectively. These values were lower than the *R^2^* values processed with DWT (Figure 6), indicating that the calibration curves fit better after the DWT processing. Thus, the data obtained after processing were better suited to quantitative analysis, with the DWT method also improving the stability and accuracy of the data. A high degree of linearity was found between the HM contents in the soil and the XRF spectral characteristic peak intensities from measurements within the appropriate range. A model was established to determine a calibration curve for the instrument.

### 3.3. Detection Results and Detection Limits

**Figure 7:**
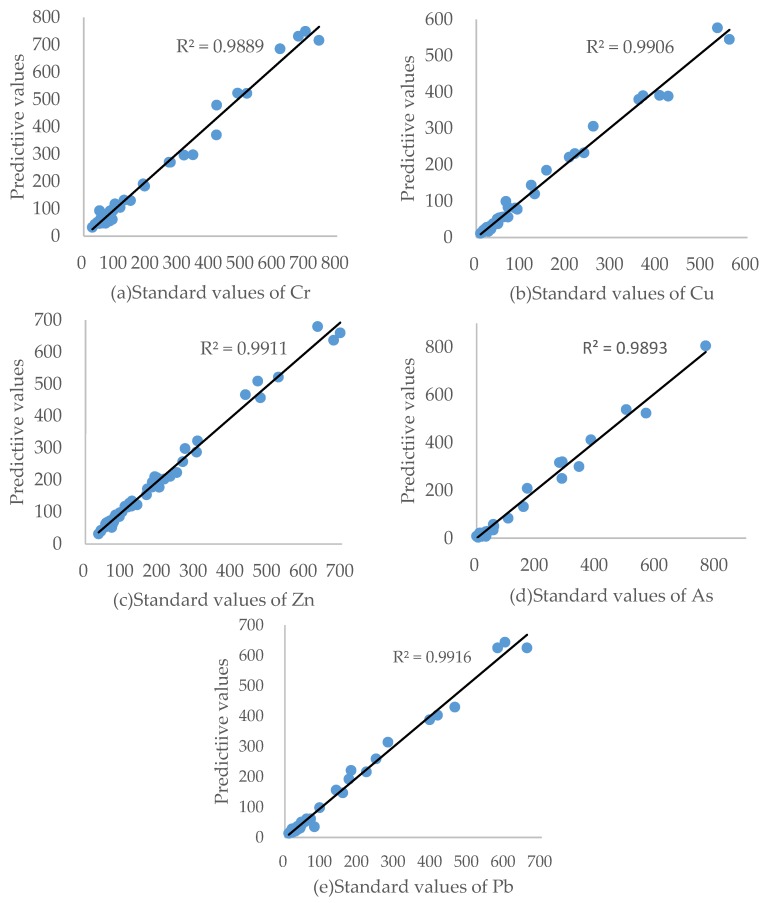
Relationship between standard values measured by chemical analysis and predictive values detected by XRF spectrometer for Cr (**a**), Cu (**b**), Zn (**c**), As (**d**) and Pb (**e**). Spectral data were pre-processed with DWT.

The standard values of HMs in soil samples were tested by chemical methods, the results were compared to the XRF testing results with original data and processed data using DWT (Figure 7). This showed that, within their standard deviation range, the detected values were closer to the standard values, indicating that the calibration line was sufficiently accurate. A confirmatory test was performed according to the method described in 2.3. The instrument’s qualitative detection limit (QDL) is three times the standard deviation of the silicon dioxide (blank), while the quantitative detection limit (QNDL) is ten times the standard deviation of the blank. The detection limits of the raw spectra and the spectra processed by DWT were calculated. The results are shown in Table 4. The table shows that both the QDL and QNDL values were considerably reduced. Thus, the DWT method has potential for practical applications.

## 4. Conclusions

Denoising and baseline correction by the DWT method as a pre-processing procedure show good effectiveness for handling results of XRF spectroscopy. The Coiflet 3 wavelet base was chosen as an optimized filter for DWT. Good denoising effects were achieved at decomposition level 3 and an approximation of the baseline was achieved at decomposition level 9. A comparison of the calibration curves and detection limits between the raw spectral data and processed spectral data with DWT was performed. The results show that the accuracy is higher and the detection limits are lower for the processed data, which means that better modeling results can be obtained using DWT. The portable XRF spectrometer is capable of rapid detection of Cr, Cu, Zn, As, and Pb in soil within 100 s. The detection limits of the instrument were below the national secondary standard level (Environmental Quality Standard for Soils, GB15618-1995) in processed spectra, and the instrument showed good precision and accuracy, indicating potential for use in rapid in-situ screening of HM contamination in soil.

## Figures and Tables

**Table 1:** Characteristic X-ray line, peak position and absorption band of five HMs.

HMs	X-ray Line for Analysis	Peak Position/keV	Corresponding Channel	Absorption Band/keV
Cr	Kα	5.414	836	5.399–5.429
Cu	Kα	8.047	1243	8.032–8.062
Zn	Kα	8.638	1334	8.623–8.653
As	Kα	10.543	1628	10.528–10.598
Pb	Lβ	12.611	1948	12.595–12.625

**Table 2:** Denoising effects evaluated with different WBs.

WB	SNR	MSE	H	*α*
coif2	103.85	528.59	0.1120	45.46
coif3	110.57	519.53	0.1241	37.85
coif4	110.09	519.36	0.1207	39.09
coif5	98.66	516.30	0.1170	44.72
db5	99.46	531.07	0.1185	45.05
db6	100.62	524.08	0.1232	42.53
db7	99.65	517.64	0.1140	45.58
db8	111.44	518.96	0.1218	38.25
db9	104.54	516.39	0.1076	45.93
db10	103.59	520.54	0.1172	42.87
sym5	105.45	520.84	0.1136	43.37
sym6	107.66	519.21	0.1099	43.88
sym7	95.79	517.49	0.1183	45.68
sym8	97.53	519.33	0.1121	47.49

**Table 3:** Results of different decomposition levels with coif 3.

Decomposition Level	SNR	MSE	H	*α*
3	110.57	519.53	0.1241	37.85
4	107.46	603.68	0.1044	53.81
5	104.98	686.08	0.0584	111.84
6	97.52	743.46	0.0487	156.68
7	113.44	779.65	0.0237	290.31
8	98.06	798.07	0.0280	290.83
9	141.54	807.36	0.0313	182.44
10	92.70	815.65	0.0349	252.04

**Table 4:** Instrument detection limits (*n* = 11).

Detection Limits	Cr	Cu	Zn	As	Pb
QDL	11.34	9.33	7.59	7.25	11.67
QNDL	37.80	31.10	25.31	24.16	38.91
WT-QDL	2.22	6.13	3.87	4.52	5.28
WT-QNDL	7.39	20.43	12.90	15.08	17.61
National level	90	35	100	20	35
